# Spatial distribution and characteristics of HIV clusters in Ethiopia*

**DOI:** 10.1111/tmi.13356

**Published:** 2020-01-12

**Authors:** Roger Ying, Lelisa Fekadu, Bruce R. Schackman, Stéphane Verguet

**Affiliations:** ^1^ Yale School of Medicine Yale University New Haven CT USA; ^2^ Department of Global Health and Primary Care University of Bergen Bergen Norway; ^3^ Federal Ministry of Health Addis Ababa Ethiopia; ^4^ Department of Healthcare Policy and Research Weill Cornell Medical College Cornell University New York NY USA; ^5^ Department of Global Health and Population Harvard T.H. Chan School of Public Health Boston MA USA

**Keywords:** HIV, spatial distribution, hot spots, sub‐Saharan Africa, Ethiopia, risk factors, VIH, distribution spatiale, points chauds, Afrique subsaharienne, Ethiopie, facteurs de risque

## Abstract

**Objectives:**

Ethiopia’s HIV prevalence has decreased by 75% in the past 20 years with the implementation of antiretroviral therapy, but HIV transmission continues in high‐risk clusters. Identifying the spatial and temporal trends, and epidemiologic correlates, of these clusters can lead to targeted interventions.

**Methods:**

We used biomarker and survey data from the 2005, 2011 and 2016 Ethiopia Demographic and Health Surveys (DHS). The spatial‐temporal distribution of HIV was estimated using the Kulldorff spatial scan statistic, a likelihood‐based method for determining clustering. Significant clusters (*P *< 0.05) were identified and compared based on HIV risk factors to non‐cluster areas.

**Results:**

In 2005, 2011 and 2016, respectively, 219, 568 and 408 individuals tested positive for HIV. Four HIV clusters were identified, representing 17% of the total population and 43% of all HIV cases. The clusters were centred around Addis Ababa (1), Afar (2), Dire Dawa (3) and Gambella (4). Cluster 1 had higher rates of unsafe injections (4.9% *vs.* 2.2%, *P* < 0.001) and transactional sex (6.0% *vs.* 1.6%, *P* < 0.001) than non‐cluster regions, but more male circumcision (98.5% *vs.* 91.3%, *P* < 0.001). Cluster 2 had higher levels of transactional sex (4.9% *vs.* 1.6%, *P* < 0.01), but lower levels of unsafe injections (0.8% *vs.* 2.2%, *P* < 0.01). Cluster 3 had fewer individuals with> 1 sexual partner (0% *vs.* 1.7%, *P* < 0.001) and more male circumcision (100% *vs.* 91.3%, *P* < 0.001). Cluster 4 had less male circumcision (59.1% *vs.* 91.3%, *P* < 0.01).

**Conclusions:**

In Ethiopia, geographic HIV clusters are driven by different risk factors. Decreasing the HIV burden requires targeted interventions.

## Abstract (fr)

**Objectifs:**

La prévalence du VIH en Ethiopie a diminué de 75% au cours des 20 dernières années avec l’implémentation du traitement antirétroviral, mais la transmission du VIH se poursuit dans les grappes à haut risque. L'identification des tendances spatiales et temporelles et des corrélations épidémiologiques de ces grappes peut mener à des interventions ciblées.

**Méthodes:**

Nous avons utilisé des biomarqueurs et des données d'enquête provenant des Surveillances Démographiques et de Santé (SDS) en Ethiopie de 2005, 2011 et 2016. La distribution spatiotemporelle du VIH a été estimée à l'aide de la statistique de balayage spatial de Kulldorff, une méthode basée sur la probabilité de déterminer des regroupements. Des grappes significatives (*P* < 0.05) ont été identifiées et comparées sur base des facteurs de risque du VIH dans les zones sans regroupements.

**Résultats:**

En 2005, 2011 et 2016, respectivement, 219, 568 et 408 personnes ont été testées positives pour le VIH. Quatre grappes de VIH ont été identifiées, représentant 17% de la population totale et 43% de tous les cas de VIH. Les grappes étaient centrées sur Addis‐Abeba (1), Afar (2), Dire Dawa (3) et Gambella(4). La grappe 1 avait des taux plus élevés d'injections à risque (4,9% contre 2,2%, *P* < 0.001) et de rapports sexuels transactionnels (6,0% contre 1,6%, *P* < 0.001) que les régions sans regroupement, mais plus de circoncisions masculines (98,5% contre 91,3%, *p* <0,001). La grappe 2 avait des taux plus élevés de rapports sexuels transactionnels (4,9% contre 1,6%, *P* < 0.01), mais des taux inférieurs d'injections à risque (0,8% contre 2,2%, *P* < 0.01). La grappe 3 avait moins d'individus avec >1 partenaire sexuel (0% contre 1,7%, *P* < 0.001) et plus de circoncisions masculines (100% contre 91,3%, *P* < 0.001). La grappe 4 avait moins de circoncisions masculines (59,1% contre 91,3%, *P* < 0.01).

**Conclusions:**

En Ethiopie, les grappes géographiques du VIH sont guidées par différents facteurs de risque. La réduction de la charge du VIH nécessite des interventions ciblées.

## Introduction

The last twenty years have seen a dramatic increase in quality of life for people living with HIV. Improved efficacy and side effect profiles of antiretroviral therapy (ART) have led to similar life expectancies between people living with and without HIV in high‐, middle‐, and low‐income countries 1, 2. In addition to increasing life expectancy, providing ART early in the course of HIV decreases the risk of HIV transmission by over 90% 3. For men at risk of acquiring HIV, male circumcision decreases the risk of acquiring HIV by 60% and provides a permanent means to reducing HIV transmission 4. Finally, providing ART as pre‐exposure prophylaxis (PrEP) also decreases the risk of HIV acquisition by over 80% 5.

By expanding the use of ART and male circumcision, Ethiopia has reduced its HIV prevalence from nearly 4% in 1998 to approximately 1% today 6. Policies such as providing free ART for eligible patients and establishing a national HIV governing body to coordinate HIV efforts have contributed to the declining HIV burden 7, 8. As a result, by 2017, over 60% of people living with HIV were on ART, and over 90% of adult males were circumcised 9. However, the reductions in HIV prevalence have not been uniform across the country. Regions such as Tigray and Amhara have seen over 50% reductions in HIV prevalence from 1999 to 2016, while others such as Harari and Afar have seen substantially smaller reductions 10, 11.

With a heterogeneous HIV burden and a decreasing national HIV prevalence, Ethiopia’s epidemic is primarily concentrating in certain populations. Previously a generalised epidemic, it is now considered a mixed epidemic according to WHO classifications 12. The greatest variability in HIV is between urban and rural areas, with the most recent Ethiopia Demographic and Health Survey estimating an HIV prevalence of 2.9% in urban regions and 0.4% in rural ones 11. Further reductions in HIV incidence will require a nuanced approach that addresses the risk factors specific to geographic clusters of continued HIV burden. Many risk factors have previously been identified, including injection drug use 13, multiple sex partners 14, transactional sex 14 and unskilled manual labour 15. Furthermore, these factors vary by region and stage of HIV epidemic 14.

In light of the heterogeneity in risk profiles among countries and regions, previous modelling studies have shown the potential impact of targeted interventions in these populations, demonstrating significantly greater cost‐effectiveness for targeted strategies 16, 17. Ethiopia would benefit from such a strategy focused on the regional epidemics in specific clusters. By identifying factors associated with HIV transmission in each cluster, interventions can be targeted to effectively and efficiently reduce the HIV burden in those regions and thus in Ethiopia as a whole. This study aims to identify geographic clusters of HIV in Ethiopia and determine the biological and behavioural correlates for each of these HIV clusters.

## Methods

HIV and global positioning system (GPS) data from the 2005, 2011 and 2016 Ethiopia Demographic and Health Surveys (DHS) were analysed using spatial statistics to assess for geographic clustering. The geographic clusters were then evaluated for biological and behavioural correlations.

### Data sources

We used data from the 2005, 2011 and 2016 Ethiopia DHS, while excluding the 2000 Ethiopia DHS due to lack of biologic HIV data. The data collection and de‐identification processes have been described previously and are summarised here 18, 19, 20. Houses were selected using a two‐stage sample clustering procedure, wherein enumeration areas (EAs) were delineated and randomly selected, and then households within selected EAs were also enumerated and randomly selected for surveying. Enumeration is repeated for each survey such that the entire nationally registered population is represented for every survey. Thus, the survey does not differentiate between incident and prevalent cases. Women aged 15–49 years and men aged 15–59 years within the household were eligible to complete a questionnaire and test for HIV with dried blood spots. The longitude and latitude of each EA were also recorded. Confidentiality was maintained by randomly displacing households by up to 2 km for urban households and up to 5 km for rural households, with a random 1% of rural households being displaced by up to 10 km. The coordinates of the EA in which the final displaced household lies were recorded.

### Cluster analysis

Clusters of HIV cases were estimated using the SaTScan software (v9.6, http://www.satscan.org). The method has been described previously and is summarised here 21, 22. In the case of HIV, the Kulldorff method models HIV cases as Bernoulli trials. Circular areas of variable radii are centred around each GPS coordinate, and the HIV prevalence inside and outside the area is calculated. The radii range from the shortest distance between two individuals with HIV and a radius that encompasses one‐half of the study population. For each circular area, the likelihood of the alternative hypothesis that the HIV prevalence inside the area is greater than the prevalence outside the area is compared to the null hypothesis that the two prevalences are equal. To assess temporal trends of clusters, equivalent circular areas are used for each survey year (2005, 2011, 2016) and the prevalence of HIV inside and outside the circles is compared among those years. This process is repeated 9,999 times while varying the radius and location of the circle, and clusters that are statistically significant (*P* < 0.05) are saved. We limited the maximum tested cluster size to contain up to 25% of the population in order to include sparsely populated regions that may have similar risk factor profiles. Finally, we mapped the locations of clusters for each survey year and identified those clusters that were persistent throughout the three survey years as our final clusters.

### Cluster correlations

We assessed the biological and behavioural correlates for significant clusters. The biological and behavioural correlates we included were the proportion of respondents reporting ever having unsafe injections 13, having more than one sexual partner 14, engaging in sexual intercourse in exchange for money or gifts 14, working in unskilled manual labour 15 and being circumcised (only for men) 4. Responses were excluded if they were reported as ‘Not applicable’, ‘Don’t know’ or ‘Missing’. These factors were only evaluated in the 2016 DHS data with the goal of determining actionable differences. Descriptive statistics for all variables were calculated for each cluster and compared between each cluster and all non‐cluster areas using two‐sided Student’s t‐tests. Due to the few HIV cases in several areas, comparisons between clusters and non‐cluster areas included all individuals in a given cluster rather than limiting to only those living with HIV. Statistical significance was defined at the level of *P* < 0.01. All values reflect the sampling weights as described by the DHS 23. All analyses were conducted in the R programming language (v3.5.1).

## Results

**Figure 1:**
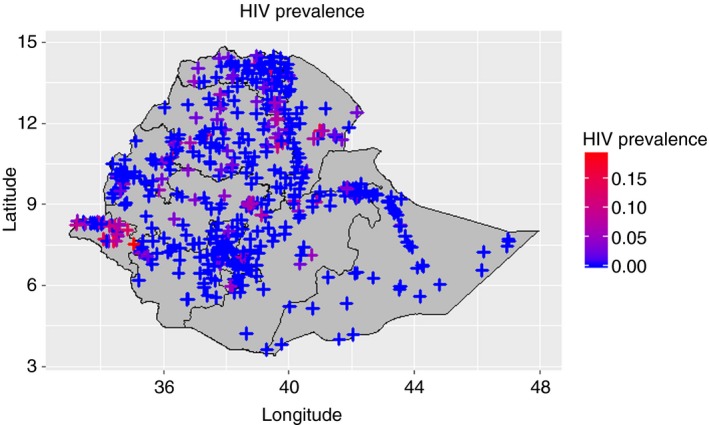
Map of HIV prevalence in Ethiopia. Each ‘+’ represents an enumeration area, with the colour representing the HIV prevalence in each EA. Blue represents low HIV prevalence, and red represents high HIV prevalence.

The data sets included 11,383 participants in DHS conducted in 2005; 29,812 participants in 2011; and 26,753 participants in 2016. In 2005, 219 individuals tested positive for HIV; in 2011, it was 568; and in 2016, it was 408. Given the de‐identification process, survey participants were associated with 535 unique GPS coordinates in 2005, with 596 in 2011 and with 643 in 2016 (Figure 1).

**Figure 2:**
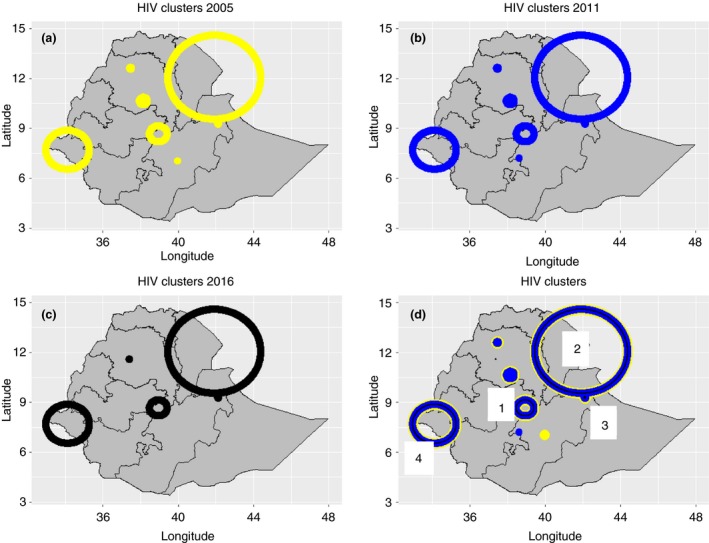
Maps of HIV clusters in Ethiopia. Circles represent clusters in (a) 2005, (b) 2011 and (c) 2016, with the size indicating cluster size and colour indicating the year in which the cluster existed. Yellow represents 2005, blue represents 2011, and black represents 2016. Only four clusters are present in every year and are labelled 1‐4 (d).

In 2005, 2011 and 2016, there were seven, seven and five total clusters identified, respectively, four of which were consistent throughout all three surveys (Figure 2). The consistent clusters (henceforth called ‘clusters’) were centred around Addis Ababa, Afar, Dire Dawa, and Gambella, and their characteristics are summarised in Table 1. In total, these clusters represented approximately 17% of the total population, but accounted for over 43% of the HIV population in 2016. Whereas the national HIV prevalence was less than 1%, the HIV prevalence in the four clusters was approximately 3%. The clusters that existed in 2005 and 2011 but not in 2016 represented less than 1% of the people living with HIV in Ethiopia.

**Table 1:** Characteristics of HIV clusters in 2016 survey

Clusters	HIV prevalence % (n/N)	Proportion of survey respondents % (n/N)	Proportion of HIV‐positive survey respondents % (n/N)
1 (Addis Ababa)	2.6 (54/2054)	10.6 (2369/22295)	23.7 (54/228)
2 (Afar)	3.7 (41/1094)	5.4 (1209/22295)	18.0 (41/228)
3 (Dire Dawa)	5.1 (2/39)	0.2 (43/22295)	0.9 (2/228)
4 (Gambella)	4.3 (2/47)	0.2 (53/22295)	0.9 (2/228)
1, 2, 3, 4 (Total)	3.1 (99/3234)	16.5 (3674/22295)	43.4 (99/228)
Non‐cluster areas (Remaining areas)	0.6 (128/21785)	83.5 (18621/22295)	56.6 (129/228)

Differences in biological and behavioural factors in the populations living in clusters and non‐cluster areas in 2016 are shown in Table 2. Cluster 1, centred on Addis Ababa, had higher levels of individuals reporting unsafe injections (4.9% *vs.* 2.2%, *P* < 0.001) and transactional sex (6.0% *vs.* 1.6%, *P* < 0.001) than non‐cluster areas, but also had higher levels of male circumcision (98.5% *vs.* 91.3%, *P* < 0.001). Cluster 2, centred on Afar, also had higher levels of transactional sex (4.9% vs 1.6%, *P* < 0.01), but lower levels of unsafe injections (0.8% *vs.* 2.2%, *P* < 0.01). Cluster 3, centred on Dire Dawa, had lower levels of multiple sex partners (0.0% *vs.* 1.7%, *P* < 0.001) and transactional sex (0.0% *vs.* 1.6%, *P* < 0.001), but higher levels of reporting unsafe injections, although the difference was not statistically significant. Finally, Cluster 4, centred on Gambella, had significantly lower levels of unsafe injections (0.0% *vs.* 2.2%, *P* < 0.001) and high‐risk occupations (0.0% *vs.* 2.5%, *P* < 0.005), but also significantly lower levels of male circumcision (59.1% *vs.* 91.3%, *P* < 0.01). Although no individual cluster had higher levels of high‐risk occupations, when taken together, all clusters had a higher level of high‐risk occupations (3.5% *vs.* 2.5%, *P* < 0.01).

**Table 2:** Biological and behavioural correlates between clusters and non‐cluster areas in 2016

Clusters	Ever unsafe injection, % (n/N)	>1 sex partner in last year, % (n/N)	Transactional sex in last year, % (n/N)	High‐risk occupation, % (n/N)	Male circumcision, % (n/N)
1 (Addis Ababa)	4.9** (46/932)	2.1 (50/2368)	6.0** (134/2224)	3.4 (54/1590)	98.5** (995/1010)
2 (Afar)	0.8* (4/520)	1.3 (16/1211)	4.9* (40/1276)	3.5 (34/968)	88.6 (496/560)
3 (Dire Dawa)	8.3 (1/12)	0** (0/43)	0** (0/38)	3.0 (1/33)	100** (19/19)
4 (Gambella)	0** (0/20)	8.7 (2/23)	5.4 (3/56)	0** (0/42)	59.1* (13/22)
1, 2, 3, 4	3.4 (50/1483)	1.9 (68/3674)	4.9** (178/3596)	3.5* (91/2635)	94.5** (1524/1613)
Remaining area	2.2 (152/7044)	1.7 (422/24172)	1.6 (393/24298)	2.5 (485/19739)	91.3 (9883/10825)

*P < 0.01; **P < 0.001.

## Discussion

The HIV epidemic in Ethiopia is characterised by geographically distinct clusters of HIV. Using DHS data, we found multiple geographical areas comprising a disproportionately high number of people living with HIV. In 2005, the first Ethiopia DHS survey to test for HIV, there were seven geographic clusters of HIV. By 2016, there were five clusters, with four of them having been identified as clusters in every survey year. These areas are epidemiologically distinct and can be characterised by certain biological and behavioural risk factors for HIV when compared to areas that are not HIV clusters. For example, the first region had significantly higher levels of unsafe injections and transactional sex. The second cluster also had higher levels of transactional sex, but no other risk factors. The third cluster was notable for not having any statistically significant risk factors for HIV, although it had high levels of people reporting unsafe injections, which did not reach statistical significance. Finally, the fourth cluster was characterised by its low proportion of men who are circumcised.

The results are consistent with our understanding of HIV in Ethiopia. The presence of clusters around urban regions such as Addis Ababa, and transportation corridors such as Afar which lies along the Addis Ababa‐Djibouti route, has been described before 24. The low levels of circumcision in Gambella are also consistent with its demographics of predominantly Protestant and Catholic populations rather than Orthodox or Muslim, who regularly practice religious circumcision 8, 25, 26.

However, our epidemiologic clustering is different from prior studies of clustering that have incorporated, among other techniques, genetic sequencing, which has the advantage of being real time, but can be a costly strategy for defining and intervening on HIV clusters 27. The method used here of investigating HIV clustering using geographic data has only recently been used, but next to similar studies, our results are comparable with important differences. Cuadros et al. 28 used a similar method to identify high and low HIV prevalence clusters in multiple African countries, including Ethiopia in 2011, and found clusters of high HIV prevalence that overlapped with our current findings. However, the exact size and location of the identified clusters differed likely due to differences in our maximum allowed cluster size. Cuadros et al. used a radius‐based maximum cluster size (100 km) and identified five significant clusters, unlike our population proportion‐based maximum (25% of total population) which identified six significant clusters. We believe that our population proportion‐based maximum accounts for large, sparsely populated regions that may have similar HIV epidemic patterns. Lakew et al. also identified HIV clusters in Ethiopia in 2011 and found two clusters, both of which overlapped with our study 29. Similarly, differences in the number of clusters are likely due to their maximum of 50% of the population compared to ours of 25%, leading to two clusters, whereas we found five. They also identified national characteristics that were associated with HIV. In crude analyses, unskilled workers, multiple sex partners and urban residence were associated with HIV. Multiple sex partners and urban residence continued to be associated with HIV in adjusted analyses, consistent with our findings.

This study used a large, nationally representative survey data set for analyses, but there are several potential shortcomings with the data. First, the scope of survey questions: for example, risk factors for HIV related to occupation and injection drug use are broad categories that may be interpreted subjectively, such as the response ‘unskilled manual labour’ for occupation, or the acknowledgement of ‘unsterile injections’ for intravenous drug use. Most importantly, the DHS also did not ask about ART use, and thus, this study could not assess how ART coverage correlated with persistence of HIV clusters. Our study also had small sample sizes, particularly in Clusters 3 (Dire Dawa) and 4 (Gambella), resulting in differences between clusters that did not reach statistical significance. Finally, the spatial analysis was limited by our use of the Kulldorff spatial statistic. A prior study comparing multiple methods for detecting clustering and identifying clusters found overall consistency among methods, but that for cluster identification, results were impacted by the size of the study area and the population and size of the control group 30. However, all of our analyses consistently used the same sample for comparison testing, decreasing the influence of sample size variability. The prior study also identified the Kulldorff spatial statistic’s use of circular clusters as potentially limiting 30. However, several prior studies used the Kulldorff method and its use here allows for comparison among studies 29, 31.

The understanding of regional differences in HIV risk factors in Ethiopia has been known since early in Ethiopia’s HIV epidemic, with a particular focus on the urban/rural divide 8. In addition to Ethiopia, other studies have identified spatial variability in countries with declining HIV prevalence such as Tanzania, Malawi, Kenya and Zimbabwe 31. Global policy organisations are now proposing nuanced responses to heterogeneous HIV epidemics, including UNAIDS’ ‘Know Your Epidemic’ campaign 32. In Ethiopia, recent efforts have focused on mapping geographic ‘hotspots’ to target community mobilisation and increased HIV service delivery to at‐risk populations, with a particular focus on sex workers, truck drivers, mobile labourers and household members of people living with HIV 33. This ‘catch‐up campaign’ strategy aims to efficiently identify individuals living with HIV. Therefore, studies such as the current spatial analysis are important for identifying which subsequent interventions are most relevant to each hotspot and can cost‐effectively reduce the HIV burden. Future mathematical modelling studies could use the new understanding of heterogeneous HIV epidemics to create nuanced approaches to treating HIV 16, 34, 35.

HIV prevalence in Ethiopia has decreased dramatically but remains elevated in clusters around the country. These remaining clusters have unique risk factors that drive their continued HIV transmission. This framework of locating HIV clusters and identifying their unique risk factors can aid policymakers in developing nuanced and cost‐effective HIV treatment and prevention strategies.
